# Drug Manipulation in Pediatric Care: A Scoping Review of a Widespread Practice Signaling Systemic Gaps in Pharmaceutical Provision

**DOI:** 10.3390/pharmacy14010002

**Published:** 2025-12-24

**Authors:** Charlotte Vermehren, Laura Giraldi, Sarah Al-Rubai, Ida M. Heerfordt, Yasmine Merimi, Rene Mathiasen, Anette Müllertz, Jon Trærup Andersen, Susanne Kaae, Christina Gade

**Affiliations:** 1 Department of Clinical Pharmacology, Copenhagen University Hospital—Bispebjerg and Frederiksberg, 2400 Copenhagen, Denmark; 2 Capital Region Pharmacy, Copenhagen University Hospital, 2400 Copenhagen, Denmark; 3 Department of Drug Design and Pharmacology, University of Copenhagen, 2200 Copenhagen, Denmark; 4 Department of Clinical Medicine, University of Copenhagen, 2200 Copenhagen, Denmark; 5 Department of Pediatrics, Copenhagen University Hospital, 2100 Copenhagen, Denmark; 6 Department of Pharmacy, Faculty of Health and Medical Sciences, University of Copenhagen, 2200 Copenhagen, Denmark

**Keywords:** pediatric pharmacotherapy, drug manipulation, age-appropriate formulations, off-label use, formulation gaps, medication safety

## Abstract

Background: Pediatric patients often receive medicines manipulated from adult formulations due to a lack of age-appropriate products. While such practices are clinically routine, they may reflect deeper systemic deficiencies in pediatric pharmacotherapy. Objective: This scoping review aimed to map the prevalence, definitions, and types of pediatric drug manipulation and to conceptualize manipulation as an indicator of structural gaps in formulation science, regulation, and access. Methods: A systematic search of PubMed (January 2014–July 2024) included 10 studies reporting the frequency of drug manipulation in children aged ≤18 years. Eligible studies were synthesized narratively according to PRISMA-ScR guidelines. Results: Ten studies from nine countries were included, reporting manipulation frequencies ranging from 6.4% to 62% of all drug administrations and up to 60% at the patient level. Manipulated formulations most commonly included oral solid doses, altered through dispersing, splitting, or crushing. Definitions and methodologies varied considerably. The findings revealed five recurring structural gaps: limited pediatric formulations, inconsistent regulatory implementation, lack of standardized definitions and guidance, insufficient evidence on manipulation safety, and inequitable access across regions. Conclusion: Manipulation of finished dosage forms for use in children is a widespread, measurable phenomenon reflecting systemic inadequacies in formulation development, regulation, and access. Recognizing manipulation as a structural indicator may guide policy, innovation, and equitable pediatric pharmacotherapy worldwide.

## 1. Introduction

Children constitute a significant proportion of healthcare recipients globally; however, the majority of approved medicines are still primarily developed, tested, and formulated for adults [1]. Despite regulatory incentives from the European Medicines Agency (EMA) and U.S. Food and Drug Administration (FDA) to promote pediatric-appropriate drug formulations, many medicines still lack commercially available pediatric formulations. Economic and market constraints hinder the development of child-specific formulations, especially for off-patent drugs, meaning that regulatory encouragement alone does not ensure availability of safe, evidence-based pediatric products [2].

As a consequence, pediatric patients often receive drugs in dosages and formulations that are not suited to their physiological, developmental, or therapeutic needs [3,4]. This fundamental mismatch creates a persistent dependency on drug manipulation—the alteration of licensed dosage forms through splitting, crushing, dispersing, or compounding to achieve age-appropriate doses [5].

The pediatric population, as defined in regulatory frameworks, includes individuals from birth to 17 years and is usually stratified into age-based subgroups. The physiological variability of children, including differences in organ maturity, metabolic capacity, and body composition, increases their vulnerability to dosing inaccuracies and adverse drug reactions [6,7,8]. Manipulated medicines may exhibit unpredictable stability, bioavailability, and pharmacokinetics, particularly for drugs with narrow therapeutic windows or modified-release mechanisms [9,10,11,12,13,14]. Consequently, manipulation can lead to both underdosing and overdosing, compromising efficacy and safety across a range of clinical settings [15].

Despite these risks, drug manipulation remains an intrinsic component of pediatric pharmacotherapy. Healthcare professionals routinely alter adult formulations to accommodate pediatric needs, often without manufacturer guidance or evidence-based support [3,6,16]. Studies have shown that tablet splitting, for example, can produce significant dose variability [15], while dispersing or crushing coated or sustained-release preparations can alter absorption and pharmacodynamic profiles [17]. These practices are often undertaken out of necessity rather than choice.

Traditionally, drug manipulation has been viewed as a local workaround—a pragmatic response to the lack of suitable pediatric medicines. However, its ubiquity and persistence across health systems suggest a deeper systemic problem.

Understanding the scope and variation of these practices is therefore critical not only for clinical safety but also for identifying underlying structural barriers to optimal pediatric care. By mapping the prevalence, definitions, and characteristics of pediatric drug manipulation across contexts, this scoping review aims to conceptualize manipulation as a structural metric—a lens through which gaps in formulation science, regulation, and access can be revealed. In this context, a structural metric refers to a measurable phenomenon that reflects underlying system-level properties rather than isolated clinical behaviors. In the context of pediatric pharmacotherapy, drug manipulation serves as such a metric because it is not simply an individual practice, but rather the visible manifestation of deeper structural constraints within health systems, pharmaceutical markets, and regulatory frameworks. Hence, pediatric drug manipulation as a structural metric refers to the use, frequency, and nature of medicine alterations as a quantifiable indicator of system-level deficiencies in the availability, regulation, guidance, and real-world accessibility of age-appropriate formulations.

The primary objective of this study is to determine the frequency of drug manipulation in pediatric care across all healthcare settings. The secondary objectives are to quantify the number of patients receiving manipulated drugs, to characterize the types and formulations of manipulated medications, and to describe the various definitions and practices of drug manipulation applied in pediatric care.

## 2. Materials and Methods

The scoping review was reported according to the Preferred Reporting Items for Systematic Reviews and Meta-Analyses extension for Scoping Reviews (PRISMA-ScR) guidelines to ensure a transparent and systematic process [18,19]. However, no formal review protocol was registered.

Eligibility criteria: Inclusion criteria were (1) studies reporting the frequency of drug manipulation in a pediatric population (years of age ≤18) and (2) published between January 2014–July 2024. Exclusion criteria were (1) studies not available in full text, (2) case reports, (3) conference abstracts, (4) a mix of pediatric and adult population (unless specific pediatric results were specifically presented), or (5) were not published in English.

Information sources and search: The search was conducted using PubMed on 18 July 2024, using the string: [(“manipulation” OR “manipulating” OR “manipulated”) AND (“children” OR “pediatric” OR “pediatric”) AND (“drug” OR “medicine”)]. The following filters were used: language: English, population: children from birth—18 years. Manual reference screening followed. Data were extracted independently by two authors and synthesized narratively due to heterogeneity.

Selection of source evidence: One of the authors (S.A.-R.) screened potential studies by reviewing their abstracts, with those meeting the inclusion criteria proceeding to full-text evaluation. To ensure accuracy, the search strategy results were reviewed twice and independently assessed by a second author (L.G.).

Data charting process: Conducted independently by the author (S.A.-R.) and subsequently reviewed in duplicate by a second author (L.G.) to confirm accuracy and consistency. Any discrepancies identified during the data extraction process were resolved through discussion between the authors.

Data items: Information collected included publication year, country, study design, study period, number of participants, number of administrations, age of participants, frequency of manipulation (dividing the number of manipulated administrations by the total number of administrations), number of patients receiving manipulated drugs (the number of patients receiving manipulated drugs divided by the total number of patients), drug formulation, type of manipulation, and definitions of manipulation.

Critical appraisal: No formal critical appraisal was performed, as the review focused on mapping literature rather than evaluating intervention effects.

Synthesis methods: Data extracted from each included study were synthesized narratively due to the considerable heterogeneity in study designs, populations, and reporting methods. The synthesis focused on summarizing key aspects related to drug manipulation in pediatric care, including its reported frequency, the characteristics of manipulated formulations, the types of manipulations performed, and the definitions or descriptions applied across studies. Findings were presented descriptively to provide an overview of current evidence and to identify patterns, gaps, and variations in reporting practices across healthcare settings.

## 3. Results

### 3.1. Study Selection

**Figure 1:**
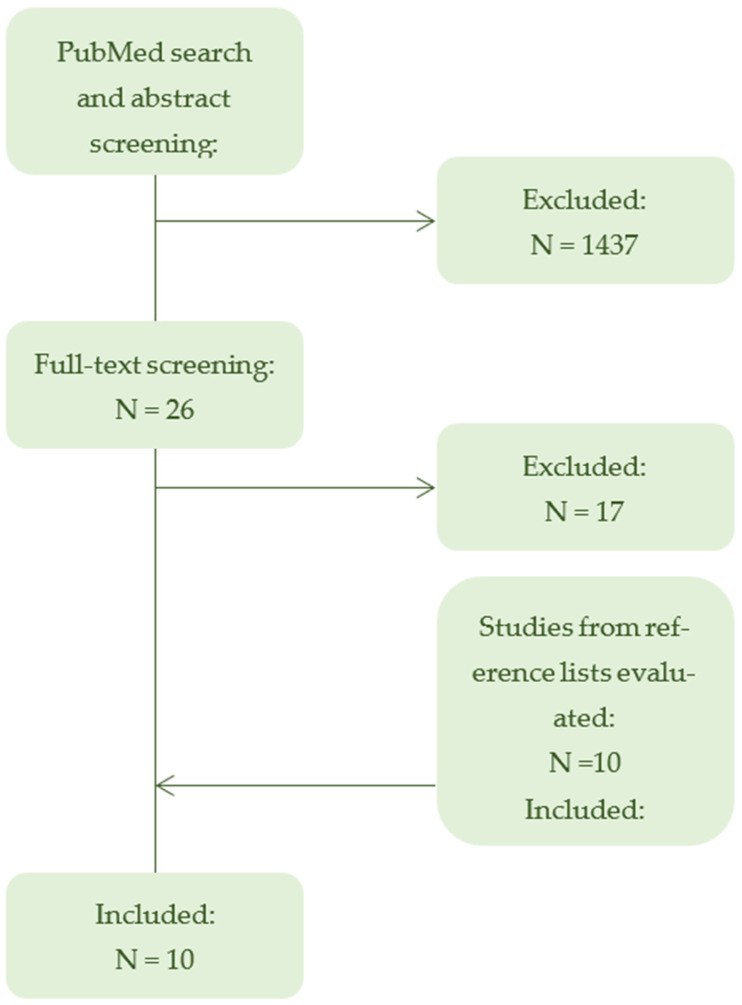
Flowchart of study selection. PRISMA-ScR flowchart summarizing the selection process of studies included in the scoping review. Of 1463 records identified through database searching, 10 studies met the inclusion criteria. After full-text screening, 17 articles were excluded, primarily due to inclusion of mixed populations comprising both adults and children, as well as articles not published in English. The flowchart illustrates the progressive exclusion of ineligible studies (->) and the identification of additional records (<-) through reference screening.

The database search identified 1463 records, of which 1437 were excluded after abstract screening (Figure 1). Twenty-six full texts were assessed for eligibility, resulting in 9 included studies. Screening of reference lists yielded 10 additional records, with one meeting the inclusion criteria, giving a total of 10 studies included in the review (Table 1). All studies explicitly examined pediatric drug manipulation as a primary research focus, reflecting a growing but fragmented body of evidence.

### 3.2. Study Characteristics

The included studies were conducted between 2013 and 2021 and published between 2016 and 2023 across nine countries (Norway, Kenya, the Netherlands, Germany, China, the United States, Sweden, Sri Lanka, and Ethiopia). All were observational, with six specifying a cross-sectional design [20,21,22,23,24]. Study durations ranged from 1 month to 2.5 years, and population ages from 0 to 18 years. Sample sizes varied markedly (19–1497 participants), and the number of drug administrations ranged from 115 to 78,366. The diversity in designs, populations, and outcome measures highlights the lack of standardized methods for quantifying and reporting pediatric drug manipulation (Table 1).

### 3.3. Frequency and Nature of Manipulation

**Figure 2:**
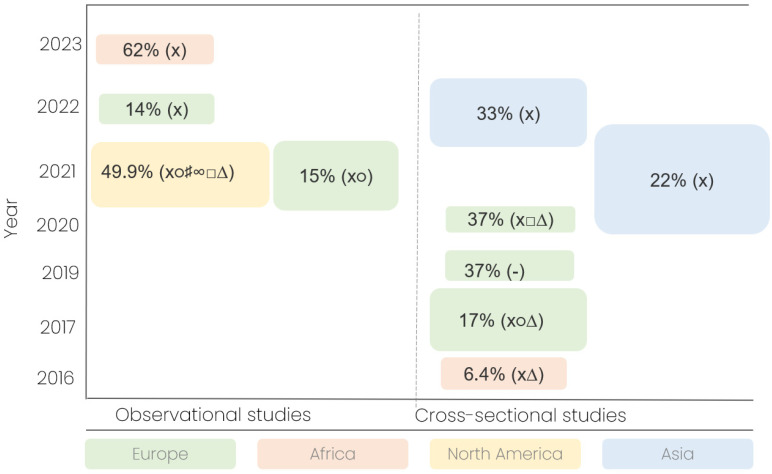
Overview of manipulation frequencies and drug formulation types in included studies. Bubble plot illustrating the reported frequencies of pediatric drug manipulation across the 10 included studies, stratified by country, study design, and publication year. Each bubble represents a single study. Bubble size is proportional to the total number of drug administrations observed in that study, with larger bubbles indicating studies with more observations. Bubble color corresponds to the continent of origin. Symbols: x, solid oral drugs; ∆, intravenous; ○, sachet; □, liquid; ♯, suppository; ∞, nebulizer; -, not provided.

Across the included studies, the frequency of pediatric drug manipulation ranged from 6.4% to 62% of all administrations (Figure 2). Two studies additionally reported patient-level frequencies—57% and 60%—indicating that more than half of the treated children received at least one manipulated medication (Table 1). The wide range of reported manipulation frequencies (6.4–62%) highlights both the lack of standardized definitions for pediatric drug manipulation and the global prevalence of gaps in age-appropriate formulations.

Manipulated dosage forms included tablets (8 studies), capsules (6), intravenous formulations (4), liquids (2), sachets (3), suppositories (1), and nebulized drugs (1). Most studies involved multiple formulation types, suggesting that manipulation practices span across dosage form categories and reflect a lack of age-appropriate formulations, rather than clinical preference.

### 3.4. Manipulation Techniques and Definitions

Reported manipulation techniques varied but most frequently included dispersing (8 studies), splitting (7), crushing (5), opening (4), fractionating (3), and mixing (1) (Table 1).

Definitions of “manipulation” differed widely [20,22,23,24,25,26,27]:

- Four studies defined it as a physical alteration of a drug.
- Two as modifications not described in the Summary of Product Characteristics.
- One as alternative administration method;
- One as adjusting a dose to achieve the exact amount prescribed.

This definitional heterogeneity complicates cross-study comparisons and demonstrates the absence of harmonized conceptual and regulatory frameworks for describing and evaluating pediatric drug manipulation.

### 3.5. Synthesis and Interpretation

Across countries and healthcare settings, the high and variable frequencies of manipulation underscore that the practice is routine yet poorly standardized in pediatric care. The data collectively reveal that manipulation functions as a proxy indicator of systemic inadequacies in pediatric drug formulation, regulation, and access.

Rather than isolated clinical adaptations, these practices reflect a structural dependence on off-label adjustments of adult dosage forms to meet pediatric needs. The inconsistencies in study definitions, outcome measures, and reporting methods further emphasize the urgent need for standardized metrics and evidence-based guidelines to ensure safe, equitable, and age-appropriate pharmacotherapy for children globally.

### 3.6. Structural Gaps in Pediatric Pharmacotherapy

To further contextualize these findings, five structural gaps are highlighted by the review:

1. Limited availability of pediatric-specific formulations;
2. Inconsistent regulatory implementation and access;
3. Lack of standardized definitions and guidance;
4. Insufficient evidence to support safe manipulation practices;
5. Inequitable access across regions.

Table 2 summarizes these structural gaps, linking the observed manipulation practices to broader systemic deficiencies.

These structural gaps illustrate that pediatric drug manipulation is not merely a clinical workaround but a measurable indicator of systemic deficiencies in formulation science, clinical pharmacotherapy, and regulation. Recognizing manipulation as a structural metric can guide targeted interventions, including the development of age-appropriate formulations, harmonized definitions, evidence-based guidance, and equitable access strategies.

## 4. Discussion

This scoping review demonstrates that drug manipulation in pediatric care is a widespread and variable practice, occurring in 6.4–62% of drug administrations and affecting up to 60% of children at the patient level (Table 1; Figure 2). Importantly, these practices extend beyond isolated clinical adaptations, reflecting systemic shortcomings in formulation availability, regulatory frameworks, clinical guidance, and healthcare infrastructure. In this context, pediatric drug manipulation can be conceptualized as a measurable indicator of broader deficiencies in pediatric pharmacotherapy (Table 2).

### 4.1. Age-Appropriateness and Formulation Challenges

Children present unique pharmacological and developmental needs, including swallowing ability, taste preferences, and excipient tolerability [28]. The lack of age-appropriate formulations forces healthcare providers to manipulate adult dosage forms using techniques such as dispersing, splitting, crushing, opening, or fractionating. Manipulations can compromise stability, bioavailability, and pharmacokinetics, particularly for drugs with narrow therapeutic windows or modified-release mechanisms [28,29]. For example, splitting a modified-release tablet may accelerate drug release, increasing the risk of toxicity, while dispersing tablets into liquids may result in variable absorption depending on the vehicle and excipient interactions [3,17]. These clinical risks underscore the need for standardized techniques and comprehensive guidance.

### 4.2. Global Disparities and Healthcare System Factors

The frequency and nature of drug manipulation vary geographically. European studies generally report lower frequencies (14–37%), likely reflecting the impact of the European Medicines Agency’s Pediatric Regulation, which mandates pediatric trials and encourages age-appropriate formulations [30]. However, even within Europe, manipulation remains common, particularly for rare indications or formulations not commercially available. Studies from low- and middle-income countries, including Kenya, Ethiopia, and Sri Lanka, report the highest and lowest manipulation rates (6.4–62%), reflecting variations in healthcare infrastructure, drug availability, and regulatory oversight (Table 1). These disparities highlight that manipulation is not merely a clinical choice but a reflection of structural inequities and systemic gaps in pediatric drug provision (Table 2).

### 4.3. Regulatory Initiatives and Limitations

Although regulatory initiatives such as the European Medicines Agency Pediatric Regulation and U.S. Best Pharmaceuticals for Children Act and Pediatric Research Equity Act legislation have increased the number of approved pediatric formulations, they do not fully eliminate the need for manipulation [30,31,32,33]. Between 2007 and 2016, 260 new pediatric formulations were approved in Europe [34], yet a substantial proportion were either not marketed or unavailable in practice [35]. Consequently, clinicians continue to rely on manipulation to achieve accurate dosing, particularly for off-patent medications or specific age groups not covered by commercial formulations [36,37].

In parallel, pharmacy compounding serves as an essential mechanism for patients whose needs are not met by commercially available formulations. The 2011 Council of Europe Resolution on quality and safety assurance for pharmacy-prepared medicines seeks to harmonize standards and reduce the quality gap between compounded and industrially manufactured products [38]. In pediatrics, where suitable formulations are often lacking, compounded preparations can provide individualized doses and age-appropriate dosage forms tailored to clinical needs. However, this practice simultaneously mitigates and exposes underlying systemic shortcomings in the availability, quality assurance, and regulation of pediatric-appropriate medicines, reinforcing the broader structural deficiencies identified in this review.

### 4.4. Manipulation as a Systemic Indicator

Conceptualizing pediatric drug manipulation as a systemic indicator allows for actionable insights. Frequent manipulation signals gaps in formulation availability, inequitable access, inadequate guidance, and potential safety risks (Table 2). It provides a measurable parameter for evaluating healthcare system performance and highlights areas for targeted intervention, including regulation, compounding practices, and formulation development. Variation in techniques and the lack of harmonized definitions complicate cross-study comparisons and indicate the urgent need for standardized metrics and evidence-based guidelines [19,28].

### 4.5. Clinical Implications and Risks

Different manipulation techniques carry distinct risk profiles. Crushing cytotoxic drugs without appropriate personal protective equipment poses health risks for healthcare providers, while inaccurate splitting or dispersing may result in under- or overdosing in patients [15,20]. Furthermore, the chemical composition of excipients, pH, and the vehicle used can alter drug solubility and bioavailability, potentially compromising therapeutic outcomes [8,28]. These factors emphasize the necessity of formal guidance, training, and standardized procedures to mitigate clinical risks.

## 5. Future Directions

To address systemic gaps highlighted by manipulation practices, several strategies are recommended:

1. Harmonization of definitions and measurement frameworks to allow reliable monitoring and international comparison.
2. Standardized reporting of manipulation frequency and techniques, including administration- and patient-level metrics.
3. Regulatory incentives and policies to encourage the development and commercialization of pediatric-appropriate formulations, particularly for rare diseases or age-specific populations.
4. Evidence-based guidance and training for healthcare providers on safe manipulation practices.
5. Innovation in pediatric formulation design, including mini-tablets, orally dispersible films, 3D-printing and taste-masked preparations, ensuring acceptability, swallowability, and stability.
6. Expanded research on excipient safety and pharmacokinetic impact of manipulated formulations in pediatric populations.

### Limitations

Heterogeneity in definitions, study designs, populations, and reporting limits comparability and generalizability. Most included studies are from Europe, with fewer data from low-resource settings, where systemic gaps may be most pronounced. The review was limited to PubMed because it provides comprehensive coverage of literature most relevant to pediatric pharmacotherapy and drug manipulation. Using PubMed allowed for a focused and efficient search of peer-reviewed, high-quality studies in English capturing the majority of relevant research. While this approach may have excluded some studies indexed in other databases or in the gray literature, it ensured a consistent and reproducible dataset for analysis. In addition, no formal critical appraisal of study quality was conducted, limiting the ability to assess risk of bias across included studies. Observational designs limit insight into underlying reasons for manipulation, and longitudinal outcomes of manipulation remain underexplored. Despite these limitations, the review provides a robust foundation for conceptualizing pediatric drug manipulation as a systemic metric and highlights the practical utility of the identified structural gaps.

## 6. Conclusions

This study shows that drug manipulation in pediatric medicine is widespread across healthcare systems globally. The types and frequency of drug manipulation, involving different types of patients, medicines and formulations, point to significant gaps in the availability of age-appropriate medicines. Variations in definitions and methods of modifying medicines confirm the lack of harmonization in practice and regulation. Our results suggest systemic deficiencies in formulation development, regulatory frameworks and healthcare infrastructure. Improving these deficiencies requires standardized definitions, validated manipulation methods, changes in legislation and innovation in pediatric medicine development. Acknowledging drug manipulation as a measurable indicator of system shortcoming may in the future inform targeted interventions that make children’s medicines safer, more effective and lead to more balance in pediatric pharmacotherapy globally.

## Figures and Tables

**Table 1:** Study characteristics and results for primary and secondary outcomes.

Year, Author, Country, Reference	Study Design	Study Period	Age Range (Years)	No. of Participants	No. of Administrations	Frequency of Manipulation	No. of Patients Receiving Manipulated Drugs	Drug Formulation	Specific Drugs	Type of Manipulation	Definition of Manipulation
2016, Nderitu, Kenya [20]	Cross-sectional study	July 2016	0–6	131	249	6.4% (16/249)	Not provided	Tablet, intravenous	Not provided	Splitting, dispersing, fractioning	Not provided
2017, Bjerknes, Norway [21]	Cross-sectional study	2013	0–17	Not provided	3070	17% (509/3070)	Not provided	Tablet, capsule, sachet, intravenous	Not provided	Splitting, crushing, dispersing	Not described in the summary of SmPC
2019, van der Vossen, Netherlands [3]	Cross-sectional study	June 2017–Jan. 2018	0–18	35	115	37% (42/115)	60% (21/35)	Not provided	Not provided	Splitting, crushing, opening, dispersing, mixing	Physical alteration of a drug
2020, Zahn, Germany [22]	Cross-sectional study	Jan. 2019–May 2019	0–18	193	640	37% (237/640)	57% (110/193)	Tablet, capsule, intravenous, liquid	Antibiotics, antiepileptics, analgetics	Splitting, dispersing, opening, fractioning	Activities to administer the medicine using an alternative strategy
2021, Zhang, China [23]	Cross-sectional study	March 2019–April 2019	0–18	Not provided	78,366	22% (17,123/ 78,366)	Not provided	Tablet, capsule	Not provided	Not provided	If the prescribed drug required manipulation to achieve the exact dose
2021, Spishock, USA [24]	Observational study	Not provided	Not provided	1497	7861	49.9% (3925/ 7861)	Not provided	Tablet, capsule, sachet, suppository, nebulizer, intravenous, liquid	Not provided	Fractioning, opening, splitting, crushing, dispersing	Physical alteration of a drug
2021, Kader, Sweden [16]	Observational study	March 2018–April 2018	18 months–18 years	131	1358	Approx. 15% (data not provided)	Not provided	Tablet, sachet, capsule	Antiepileptics, cytostatics	Not provided	Physical alteration of a drug
2022, Nadeshkumar, Sri Lanka [25]	Cross-sectional study	Jan. 2017–June 2019	0–12	663	1287	Approx. 33% (data not provided)	Not provided	Solid, oral drugs	Not provided	Crushing, dispersing	Not provided
2022, Johannesson, Sweden [26]	Observational study	Dec. 2019–Feb. 2020	1 month–11 years	19	200	14% (28/200)	Not provided	Tablet, capsule	Cytostatics	Crushing, dispersing, splitting, opening	Not described in the summary of SmPC
2023, Kasahun, Ethiopia [27]	Observational study	April 2021–June 2021	0–18	275	488	62% (303/488)	Not provided	Tablet	Not provided	Splitting, dispersing	Physical alteration of a drug

Overview of the 10 studies included in the scoping review, detailing study design, setting, population, and reported outcomes. All included studies were observational in nature, with no analytical comparison groups such as case–control or cohort designs. Studies explicitly described as cross-sectional or observational represent direct observational assessments of medication administration practices. Thus, all studies were descriptive observational studies, either cross-sectional or simple observational assessments.

**Table 2:** Structural gaps in pediatric pharmacotherapy identified through the frequency and nature of drug manipulation.

Structural Gap	Evidence from Included Studies	Implications
Limited availability of pediatric-specific formulations	Tablets (8 studies) and capsules (6 studies) most frequently manipulated; intravenous drugs (4 studies) and liquids (2 studies) also manipulated (Table 1)	Indicates persistent shortage of commercially available age-appropriate formulations, leading to frequent manipulation in clinical practice.
Inconsistent regulatory implementation and access	Manipulation rates in Europe ranged 14–37% despite EMA Pediatric Regulation; rates in LMICs varied from 6.4% (Kenya) to 62% (Ethiopia)	Highlights gaps between regulatory approval and actual access; inequities across regions suggest structural disparities in healthcare systems and supply chains.
Lack of standardized definitions and guidance	Definitions of manipulation varied across 8 studies, from physical alteration to modifications not in SmPC (Table 1)	Limits comparability across studies and hinders development of evidence-based guidelines; emphasizes need for harmonized definitions.
Insufficient evidence to support safe manipulation practices	Common manipulations included dispersing (8 studies), splitting (7), and crushing (5), yet pharmacological consequences were rarely assessed	Reflects research gaps; healthcare providers lack evidence-based guidance on safety and efficacy of manipulated medications.
Inequitable access across regions	Manipulation frequency extremes between countries: 6.4% in Kenya vs. 62% in Ethiopia	Suggests structural inequities in access to pediatric formulations, influenced by market availability, healthcare infrastructure, and socioeconomic factors.

Evidence from the included studies highlights five key areas: limited availability of age-appropriate formulations, inconsistent regulatory implementation and access, lack of standardized definitions and guidance, insufficient evidence to support safe manipulation practices, and inequitable access across regions. The table summarizes specific study findings and the implications of each structural gap for pediatric drug provision and clinical practice.

## Data Availability

The original contributions presented in this study are included in the article. Further inquiries can be directed to the corresponding author.

## References

[1] Europa Kommissionen (2013). Bedre Medicin Til Børn—Fra Idé Til Virkelighed.

[2] Paulsson M., Svendsen R.H., Andersen J.K.N., Sporrong S.K., Andersson Y., Tho I. (2025). Challenges and considerations in manipulating oral dosage forms in Peadiatric Healthcare Settings: A Narrative Review. Acta Paediatr..

[3] van der Vossen A.C., Al-Hassany L., Buljac S., Brugma J.D., Vulto A.G., Hanff L.M. (2019). Manipulation of oral medication for children by parents and nurses occurs frequently and is often not supported by instructions. Acta Paediatr..

[4] Andersson Å.C., Lindemalm S., Onatli D., Chowdhury S., Eksborg S., Förberg U. (2023). ‘Working outside the box’—An interview study regarding manipulation of medicines with registered nurses and pharmacists at a Swedish paediatric hospital. Acta Paediatr..

[5] National Institute for Health Research (2013). Manipulation of Drugs Required in Children (MODRIC)—A Guide for Health Professionals.

[6] Yaffe S. (2000). Rational Therapeutics for Infants and Children: Workshop Summary.

[7] Lim S.Y., Pettit R.S. (2019). Pharmacokinetic considerations in pediatric pharmacotherapy. Am. J. Health Syst. Pharm..

[8] Priyadharsini R., Surendiran A., Adithan C., Sreenivasan S., Sahoo F.K. (2011). A study of adverse drug reactions in pediatric patients. J. Pharmacol. Pharmacother..

[9] Richey R.H., Shah U.U., Peak M., Craig J.V., Ford J.L., Barker C.E., Nunn A.J., Turner M.A. (2013). Manipulation of drugs to achieve the required dose is intrinsic to paediatric practice but is not supported by guidelines or evidence. BMC Pediatr..

[10] Andersson Å.C., Eksborg S., Förberg U., Nydert P., Lindemalm S. (2022). Manipulated Oral and Rectal Drugs in a Paediatric Swedish University Hospital, a Registry-Based Study Comparing Two Study-Years, Ten Years Apart. Pharmaceuticals.

[11] Nunn A.J. (2003). Making Medicines That Children Can Take. https://www.scribd.com/document/479272917/Making-medicines-that-children-can-take-Nunn-2003.

[12] Verrue C., Mehuys E., Boussery K., Remon J.P., Petrovic M. (2011). Tablet-splitting: A common yet not so innocent practice. J. Adv. Nurs..

[13] Skwierczynski C., Conroy S. (2008). How long does it take to administer oral medicines to children?. Paediatr. Perinat. Drug Ther..

[14] Best B.M., Capparelli E.V., Diep H., Rossi S.S., Farrell M.J., Williams E., Lee G., van den Anker J.N., Rakhmanina N. (2011). Pharmacokinetics of lopinavir/ritonavir crushed versus whole tablets in children. J. Acquir. Immune Defic. Syndr..

[15] Richey R.H., Hughes C., Craig J.V., Shah U.U., Ford J.L., Barker C.E., Peak M., Nunn A.J., Turner M.A. (2017). A systematic review of the use of dosage form manipulation to obtain required doses to inform use of manipulation in paediatric practice. Int. J. Pharm..

[16] Kader R., Liminga G., Ljungman G., Paulsson M. (2021). Manipulations of Oral Medications in Paediatric Neurology and Oncology Care at a Swedish University Hospital: Health Professionals’ Attitudes and Sources of Information. Pharmaceutics.

[17] Nunn A., Richey R., Shah U., Barker C., Craig J., Peak M., Ford J., Turner M. (2013). Estimating the requirement for manipulation of medicines to provide accurate doses for children. Eur. J. Hosp. Pharm..

[18] Grant M.J., Booth A. (2009). A typology of reviews: An analysis of 14 review types and associated methodologies. Health Inf. Libr. J..

[19] Tricco A.C., Lillie E., Zarin W., O’Brien K.K., Colquhoun H., Levac D., Moher D., Peters Micah D.J., Horsley T., Weeks L. (2018). PRISMA Extension for Scoping Reviews (PRISMA-ScR): Checklist and Explanation. Ann. Intern. Med..

[20] Nderitu H. (2016). Prevalence of Drug Manipulation to Obtain Prescribed Dose in the Paediatric In-Patient Units in Kenyatta National Hospital. Post Graduate Diploma In Biomedical Research Methodology.

[21] Bjerknes K., Bøyum S., Kristensen S., Brustugun J., Wang S. (2017). Manipulating tablets and capsules given to hospitalised children in Norway is common practice. Acta Paediatr. Int. J. Paediatr..

[22] Zahn J., Hoerning A., Trollmann R., Rascher W., Neubert A. (2020). Manipulation of Medicinal Products for Oral Administration to Paediatric Patients at a German University Hospital: An Observational Study. Pharmaceutics.

[23] Zhang L., Hu Y., Pan P., Hong C., Fang L. (2021). Estimated Manipulation of Tablets and Capsules to Meet Dose Requirements for Chinese Children: A Cross-Sectional Study. Front. Pediatr..

[24] Spishock S., Meyers R., Robinson C.A., Shah P., Siu A., Sturgill M., Kimler K. (2021). Observational Study of Drug Formulation Manipulation in Pediatric Versus Adult Inpatients. J. Patient Saf..

[25] Nadeshkumar A., Sathiadas G., Sri Ranganathan S. (2022). Administration of oral dosage forms of medicines to children in a resource limited setting. PLoS ONE.

[26] Johannesson J., Hansson P., Bergström C.A.S., Paulsson M. (2022). Manipulations and age-appropriateness of oral medications in pediatric oncology patients in Sweden: Need for personalized dosage forms. Biomed. Pharmacother..

[27] Kasahun A.E., Sendekie A.K. (2023). Are pediatrics taking the prescribed tablet dosage form? Practices of off-label tablet modification in pediatric wards: A prospective observational study. Heliyon.

[28] Ivanovska V., Rademaker C.M.A., van Dijk L., Mantel-Teeuwisse A.K. (2014). Pediatric drug formulations: A review of challenges and progress. Pediatrics.

[29] Batchelor H.K., Marriott J.F. (2015). Paediatric pharmacokinetics: Key considerations. Br. J. Clin. Pharmacol..

[30] European Medicines Agency (2006). Formulation of Choice for the Pediatric Population.

[31] U.S. Food and Drug Administration (2018). Best Pharmaceuticals for Children Act (BPCA).

[32] U.S. Food and Drug Administration (2024). Pediatric Research Equity Act (PREA).

[33] U.S. Food and Drug Administration (2018). Food and Drug Administration Modernization Act (FDAMA) of 1997.

[34] Commission to the European Parliament and the Council (2017). 10 Years of the EU Paediatric Regulation.

[35] Tiengkate P., Lallemant M., Charoenkwan P., Angkurawaranon C., Kanjanarat P., Suwannaprom P., Borriharn P. (2022). Gaps in Accessibility of Pediatric Formulations: A Cross-Sectional Observational Study of a Teaching Hospital in Northern Thailand. Children.

[36] MacArthur R.B., Ashworth L.D., Zhan K., Parrish R.H. (2022). How Compounding Pharmacies Fill Critical Gaps in Pediatric Drug Development Processes: Suggested Regulatory Changes to Meet Future Challenges. Children.

[37] Wang S., Giannuzzi V. (2024). Paediatric formulations—Part of the repurposing concept?. Front. Med..

[38] Minghetti P., Pantano D., Gennari C.G.M., Casiraghi A. (2014). Regulatory framework of pharmaceutical compounding and actual developments of legislation in Europe. Health Policy.

